# A Case Study of Grassroots Water Conservancy Services Evaluation and Obstacle Factors Diagnosis Based on Gray Correlation-TOPSIS Model in Hunan Province, China

**DOI:** 10.3390/ijerph20010174

**Published:** 2022-12-22

**Authors:** Jie Zhang, Zihao Tang, Bin Deng, Siyan Liu, Yifei Xiang

**Affiliations:** 1 Hunan Institute of Water Resourses and Hydropower Research, Changsha 410011, China; 2 School of Hydraulic and Environmental Engineering, Changsha University of Science and Technology, Changsha 410114, China; 3 Key Laboratory of Dongting Lake Aquatic Eco-Environmental Control and Restoration of Hunan Province, Changsha 410114, China; 4 College of Artificial Intelligence, Guangxi Minzu University, Nanning 530006, China

**Keywords:** gray correlation-TOPSIS model, grassroots water conservancy, evaluation index system, barrier factor analysis

## Abstract

Based on the evaluation model of the gray correlation-TOPSIS method, this paper examines the index system of grass-roots water conservancy services in Hunan Province, China. This paper aims at the present situation of grassroots water conservancy in Hunan province, which assisted it in developing grassroots water conservancy services. The evaluation indicators include five criteria levels (institutional staffing, personnel quality, management level, public policy and service capacity) and twenty-four indicator levels. In this paper, the weight calculation method combined with an analytic hierarchy process and an entropy weight method, as well as quantitative and qualitative methods, was used to conduct an empirical study on the basic water conservancy service level of Hunan Province in 2020. The results classify grassroots water services in Hunan Province into three levels. By fitting the GDP of cities and prefectures with the comprehensive closeness, we conclude that there is considerable convergence between the grassroots water conservancy service level of Hunan Province and its local economic level. The more developed the economy, the higher the grassroots water conservancy service level. In addition, through obstacle factor analysis, the main constraints of grassroots water conservancy in various cities and prefectures are obtained. Therefore, the grassroots water conservancy service’s ability can be comprehensively improved from three aspects: serviceability, capital investment, and talent construction. This indicator system can promote the overall governance capacity of grassroots water conservancy in the future development of cities and prefectures, and it can also provide Hunan with experience and case examples for the implementation of rural revitalization.

## 1. Introduction

Water conservancy is not only an indispensable primary condition for agricultural production but also irreplaceable basic support for rural economic and social development. It is also an inseparable guaranteed system for improving rural production conditions and farmers’ living environments. The grassroots water conservancy service system is the general name of institutions and organizations that provide comprehensive services for rural farmland water conservancy construction, flood control and drought relief, rural water supply, water conservancy management, water conservancy science technology promotion, etc. In the context of China’s vigorous implementation of the rural revitalization strategy, the only way to promote the overall development of rural water conservancy and the overall revitalization of the countryside is to enhance the capacity of water conservancy services at the grassroots level. At the same time, it is also essential to improve the efficiency of water conservancy services and meet the needs of grassroots people.

Since the founding of New China, grassroots water conservancy construction has endured tortuous and complicated development thanks to changes in policies, the repetition of production systems, and the changes in the division of financial power between central and local authorities. Before the 1970s, primary water conservancy construction was focused on expanding irrigation areas and increasing grain output. Although the pace of construction slowed down in the 1980s, it significantly strengthened after the 1990s. Since China’s irrigation water is inefficient in terms of production allocation, in the current century, primary water conservancy construction is more concerned with drinking water safety and improvements in water-saving irrigation technology. In 2011, the Central Committee issued the “Decisions of the CPC Central Committee and the State Council on Accelerating the Reform and Development of Water Resources”, which is the first comprehensive deployment of water conservancy work since the founding of New China 62 years ago. Since then, grassroots water conservancy construction has paid more attention to high-quality and high-standard developments. Grassroots water conservancy is a noun with Chinese characteristics, and there is no such statement elsewhere in the world. In the 1950s, the Indian government attached great importance to water conservancy construction but focused on large-scale water conservancy projects [1]. In Japan, great importance is given to the ecological function of water conservancy projects. On the road to industrialization, Japan has gradually paid attention to the sustainable development of agriculture and the harmonious relationship between water conservancy and the environment [2]. In the New Deal period of the United States, in order to restore and revitalize the economy, the state carried out the construction of public projects focusing on water conservancy projects; this process yielded considerable achievements in the country. The evaluative research on grassroots water conservancy abroad mainly focuses on sustainable development. Rosegrant proposed that sustainable water resource management, food security, and natural environment protection should be developed together [3]. Servet et al. conducted a comprehensive evaluation of Turkey’s water resources and finally proposed to control water resources by reforming water prices [4]. Lawrence Gray conducted a water quality assessment on the Plover River in the United States, and the results showed that the water quality of the river had a huge adverse impact on the surrounding residents and organisms [5]. Darrin Magee evaluated a water conservancy project through three dimensions: species diversity, economic benefits, and geographical location [6]. Throughout the history of water conservancy development in various countries, the development of water conservancy at the grassroots level is mainly divided into two stages. Therefore, the sustainable development of water resources can be achieved by promoting the industrialization of water resources in the first stage and improving the utilization rate of water resources in the second stage.

The development of evaluation is the development of human society. At present, there are many evaluation methods, such as the analytic hierarchy process, the Delphi method, principal component analysis, and the TOPSIS method. Due to the theoretical breakthrough in the research of evaluation methods and the introduction of machine learning, neural networks, etc., there are many improved evaluation models from which to choose. Slowinski R constructed a new evaluation method based on rough set theory [7]. Jiang H combined AHP with a BP neural network to establish a new AHP-BPNN model and proved its reliability [8]. Jin proposed a new fuzzy comprehensive evaluation model (AHP-FCE) based on the improved analytic hierarchy process and applied it to the case of water resources system engineering [9]. Pontil M studied a regression support vector machine (SVMC), and this method was more accurate than the traditional BP neural network [10]. Comprehensive evaluation theory has matured and is constantly improving and innovating, but there is no qualitative standard for the quality evaluation method of construction related to the comprehensive evaluation index system.

The research on grassroots water conservancy index systems is less extensive than that of other water conservancy index systems. That said, Lu employed the “fuzzy iteration” method to estimate the evaluation, ranking and optimization selection of water conservancy project constructors [11]. Zhu established an index system positive operation for water conservancy projects on the basis of frequency analysis, theoretical analysis, and expert consultation [12]. However, the evaluation index system of water conservancy modernization is very mature. The concept of water conservancy modernization was introduced in China at the end of the last century. Qiu et al. conducted empirical research on the level of primary and secondary modernization of rural water conservancy in China’s provinces by using the theory of secondary modernization [13]. Meng et al. studied the modernization of rural water conservancy in Hubei Province by analyzing the historical process of the world’s water conservancy modernization [14]. Huang et al. built a cloud model to evaluate the water conservancy modernization of Xinyi City and proposed a predictive value consistent with the planning objectives, the findings indicated that the model was effective [15]. Zhou et al. analyzed and calculated the modernization level of water conservancy in Hangzhou by using the multi-index comprehensive evaluation method [16]. The modernization of water conservancy is relative, regional, and timely. Compared with research on the grassroots indicator system of water conservancy, this research should conform to the actual situation of Hunan Province. At the same time, the indicator system should also reflect the differences in the current grassroots service capacity of Hunan Province.

Based on the existing research in other areas, this paper focuses on the grassroots water conservancy in Hunan Province and evaluates this field for the first time in Hunan Province. This paper takes Hunan Province in 2020 as the research object and selects organizational structure, personnel quality, management level, public policy, and serviceability as evaluation indicators. Then, it uses subjective and objective comprehensive weighting methods to comprehensively calculate water conservancy index weights at the grassroots level in Hunan Province. C. L. Hwang and K Yoon established the TOPSIS method in 1981 [17,18,19], and Deng pioneered the grey system theory in 1982. Based on these two theories, this paper proposes a comprehensive analysis model [20,21,22,23,24] based on the grey correlation TOPSIS method. This model avoids the impact of subjective factors that determine the weights on the objective evaluation of the samples and then ensures the relationship structure between the original sample data. It not only reflects the impact of the differences between indicator data on the evaluation results but also avoids the condition that the weight of an indicator is too small to reflect the differences between indicators, thus increasing the reliability of the evaluation results. The model is simple in its calculation and has strong applicability in the evaluation of grassroots water conservancy. By establishing the obstacle degree model for the development of grassroots water conservancy services in Hunan Province, we provide theoretical guidance for the development of grassroots water conservancy services in various cities and prefectures, and we also provide a reference for the subsequent construction, management, and layout of grassroots water conservancy systems.

## 2. Materials and Methods

### 2.1. Selection of Indicators

Due to its vast area and significant regional differences in China, it is difficult to establish a set of indicators with wide applicability in grassroots water conservancy. Hunan Province is located in the middle of China and the middle reaches of the Yangtze River. It is named “Hunan” because most of the region is located to the south of Dongting Lake. At present, there are many indicators that reflect water conservancy at the grassroots level. If all indicators are considered, it is complex and difficult to operate. In order to verify the degree of correlation and recognition between indicators, the Delphi method feedback questionnaire was used; meanwhile, the coefficient of variation and average value were used to test the dispersion and representativeness of each indicator. 

### 2.2. Determination of Weight

The determination of weight is a key link in the evaluation process of grassroots water conservancy services in Hunan Province. The weight result is the quantitative result of indicators in the evaluation problem. Whether the weight is reasonable and correct will directly affect the reliability of the final evaluation result. Most current methods only consider subjective evaluation (such as analytic hierarchy process [25]) or objective evaluation (such as entropy weight method [26], projection pursuit method [27], etc.). In order to reflect the information contained in the data of the evaluation index and also consider the subjectivity of the experts, this study chooses the algorithm combining an entropy weight method and an analytic hierarchy process to make up for their shortcomings. The resultant weight coefficient is more reasonable than using a single calculation method.

#### 2.2.1. Calculation of Subjective Weight by Using Analytic Hierarchy Process

AHP establishes a multi-level analysis structure model and compares each element of each level in pairs; then, it normalizes the column vector of each comparison matrix and sums up the row to obtain the weight.

(1) $$A=\left(\begin{array}{cccc} 1 & a_{12} & \cdots & a_{1n}\\ a_{21} & 1 & \cdots & a_{2n}\\ \vdots & \vdots & \ddots & \vdots \\ a_{n1} & a_{n2} & \cdots & 1 \end{array}\right),i,j=1,2,\cdots ,n$$

(2) $$b_{ij}=a_{ij}/\sum_{i=1}^{n}a_{ij}$$

(3) $$B_{i}=\sum_{j=1}^{n}b_{ij}$$

(4) $$W_{i}=B_{i}/\sum_{i=1}^{n}B_{i},(i=1,2,\cdots ,n)$$

#### 2.2.2. Calculation of Objective Weight by Using the Entropy Weight Method

The entropy weight method is a representative weighting method among objective weighting methods; in addition, it follows the principle of the amount of information reflected by the variation degree of indicators, and it can largely avoid the interference of human factors.

(5) $$e_{j}=-\frac{1}{\ln n}\sum_{i=1}^{n}p_{ij}\ln(p_{ij})(j=1,2,\cdots ,m)$$

(6) $$d_{j}=1-e_{j}$$

(7) $$W_{j}=d_{j}/\sum_{j=1}^{m}d_{j}(j=1,2,\cdots ,m)$$

where $e_{j}$ is the information entropy of the evaluation index.

#### 2.2.3. Comprehensive Weighting Method

*W* represents the comprehensive weight, *W*_1_ represents the entropic weight weight, *W*_2_ represents the hierarchical analytical weight and λ preference coefficient, taking 0.5.

(8) $$W=\lambda \cdot W_{1}+(1-\lambda )W_{2}$$

### 2.3. Determination of Evaluation Model

This research adopts the decision-making model combining the grey correlation analysis method and the TOPSIS method, and the specific steps are presented in what follows.

#### 2.3.1. Build Decision Matrix

Construct a positive data matrix *X* consisting of n evaluation objects and m evaluation indicators, *X*_11_ is the evaluation indication data 1 of evaluation object 1. The specific forms are as follows:

(9) $$X=\left(\begin{array}{cccc} x_{11} & x_{12} & \cdots & x_{1j}\\ x_{21} & x_{22} & \cdots & x_{2j}\\ \vdots & \vdots & \ddots & \vdots \\ x_{i1} & x_{i2} & \cdots & x_{ij} \end{array}\right)$$

#### 2.3.2. Standardize Decision Matrix

If the dimensions of each indicator are different, it is necessary to eliminate the impact of dimensions to achieve a dimensionless form and adopt different treatment methods according to the indicator type. For very large (benefit-oriented) indicators:

(10) $${\tilde{x}}_{i}{}_{j}=\frac{x_{i}{}_{j}-\min(x_{j})}{\max(x_{j})-\min(x_{j})}$$

For very small (cost based) indicators:

(11) $${\tilde{x}}_{i}{}_{j}=\frac{\max(x_{j})-x_{i}{}_{j}}{\max(x_{j})-\min(x_{j})}$$

In the formula above, ${\tilde{x}}_{i}{}_{j}$ represents the normalized value of *x_ij_*, $\max(x_{j})$ represents the maximum value of the *j*th index, and $\min(x_{j})$ represents the minimum value of the *j*th index.

#### 2.3.3. Construct Weighted Decision Matrix

The weight is the comprehensive weight of the entropy weight method and the AHP method, and the final weight matrix *Z* is shown as follows:

(12) $$Z=\left(\begin{array}{cccc} w_{1}{\tilde{x}}_{11} & w_{2}{\tilde{x}}_{12} & \cdots & w_{n}{\tilde{x}}_{1m}\\ w_{1}{\tilde{x}}_{21} & w_{2}{\tilde{x}}_{22} & \cdots & w_{n}{\tilde{x}}_{2m}\\ \vdots & \vdots & \ddots & \vdots \\ w_{1}{\tilde{x}}_{n1} & w_{2}{\tilde{x}}_{n2} & \cdots & w_{n}{\tilde{x}}_{\mathrm{nm}} \end{array}\right)$$

#### 2.3.4. Calculate the Euclidean Distance from Each Scheme to the Positive and Negative Ideal Solutions

*Z*^+^ represents the positive ideal solution, *Z*^−^ represents the negative ideal solution, *d_i_*^+^ is the Euclidean distance from each scheme to the positive ideal solution, and *d_i_*^−^ is the Euclidean distance from each scheme to the negative ideal solution.

(13) $$Z_{}^{+}=(z_{1}^{+},z_{2}^{+},\cdots ,z_{j}^{+})=w,Z_{}^{-}=(z_{1}^{-},z_{2}^{-},\cdots ,z_{j}^{-})=0$$

(14) $$z_{j}^{+}=\max z_{ij}=w_{j},z_{j}^{-}=\min z_{ij}=0$$

(15) $$d_{i}^{+}=\sqrt{{\sum_{j=1}^{m}\left(z_{ij}-z_{j}^{+}\right)}^{2}},d_{i}^{-}=\sqrt{{\sum_{j=1}^{m}\left(z_{ij}-z_{j}^{-}\right)}^{2}}$$

#### 2.3.5. Calculate the Grey Correlation Coefficient of All Schemes with Positive and Negative Ideal Solutions

*r_i_*^+^ represents the grey correlation coefficient between each scheme and the rational solution and *r_i_*^−^ represents the grey correlation coefficient between each scheme and the negative ideal solution, where ρ represents the resolution coefficient, which is 0.5.

(16) $$r_{ij}^{+}=\frac{\underset{i}{\min}\underset{j}{\min}\left|z_{j}^{+}-z_{ij}\right|+\rho \underset{i}{\max}\underset{j}{\max}\left|z_{j}^{-}-z_{ij}\right|}{\left|z_{j}^{+}-z_{ij}\right|+\rho \underset{i}{\max}\underset{j}{\max}\left|z_{j}^{+}-z_{ij}\right|}$$

(17) $$r_{ij}^{-}=\frac{\underset{i}{\min}\underset{j}{\min}\left|z_{j}^{-}-z_{ij}\right|+\rho \underset{i}{\max}\underset{j}{\max}\left|z_{j}^{-}-z_{ij}\right|}{\left|z_{j}^{-}-z_{ij}\right|+\rho \underset{i}{\max}\underset{j}{\max}\left|z_{j}^{-}-z_{ij}\right|}$$

(18) $$r_{i}^{+}=\frac{1}{m}\sum_{j=1}^{m}r_{ij}^{+},r_{i}^{-}=\frac{1}{m}\sum_{j=1}^{m}r_{ij}^{-}$$

#### 2.3.6. Dimensionless Treatment of Euclidean Distance and Grey Correlation Coefficient

The formulas are as follows.

(19) $$D_{i}^{+}=\frac{d_{i}^{+}}{\max d_{i}^{+}},D_{i}^{-}=\frac{d_{i}^{-}}{\min d_{i}^{-}},R_{i}^{+}=\frac{r_{i}^{+}}{\max r_{i}^{+}},R_{i}^{-}=\frac{r_{i}^{-}}{\min r_{i}^{-}}$$

#### 2.3.7. Calculate the Distance between Each Scheme and the Ideal Scheme

In the formula, the values of *α* and *β* are both 0.5, which comprehensively reflects the distance between each scheme and the ideal value.

(20) $$S_{i}^{+}=\alpha D_{i}^{-}+\beta R_{i}^{+},S_{i}^{-}=\alpha D_{i}^{+}+\beta R_{i}^{-}$$

#### 2.3.8. Calculate the Comprehensive Closeness

*Q_i_*^+^ indicates the comprehensive closeness of each scheme, and the higher the value is, the better the evaluation result. On the contrary, the worse the evaluation result will be.

(21) $$Q_{i}^{+}=\frac{S_{i}^{+}}{S_{i}^{+}+S_{i}^{-}}$$

### 2.4. Barrier Factor Model

In order to establish specific factors that restrict the level of water conservancy service at the grassroots level in Hunan Province, this paper analyzes barriers to the water conservancy service The obstacle model is a diagnostic measure introduced to identify the key obstacle factors that limit the further improvement of results by using the evaluation results of the constructed indicator system. The calculation formula is as follows:

(22) $$O_{j}=\frac{w_{j}\times J_{j}}{\sum_{j=1}^{i}w_{j}\times J_{j}}\times 100\%$$

(23) $$J_{j}=1-Z_{j}$$

In the above formula, $O_{j}$ represents the obstacle degree of indicator *J*, $w_{j}$ represents the weight of indicator *j*, $J_{j}$ represents the deviation degree of indicator *J*, and $Z_{j}$ represents the standardization result of indicator *J*.

## 3. Instance Validation

### 3.1. Research Area

**Figure 1:**
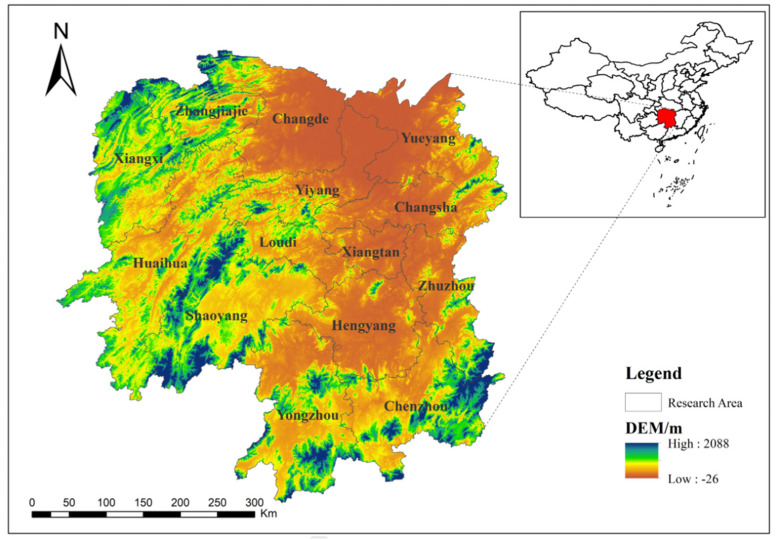
The geographical location of Hunan Province.

Hunan Province is a major water conservancy province in China (as shown in Figure 1), with 24.563 million people protected by dikes and 1733.8 thousand hectares of cultivated land. Moreover, 13,737 thousand reservoirs have been built in the province, with a total storage capacity of 54.545 billion cubic meters, and it accounts for more than 1/7 of the total number of reservoirs in the country. Basic water conservancy services in Hunan Province are led and managed by the government.

### 3.2. Data Sources

The data in this article are the sources from the selection of the evaluation index and evaluation data. The indicator data are given out to experts by means of a questionnaire using the Delphi method. The data needed for the evaluation of grassroots water conservancy services in Hunan Province are drawn from three aspects. First are the field survey statistics; second are the “statistics bulletin of water conservancy development in Hunan Province in 2020” and the “bulletin of water resources in Hunan Province in 2020”, both of which are published by the government; and third is the need to recover statistical data from the questionnaire. In total, 667 questionnaires were collected from grassroots water conservancy management organizations in cities and prefectures, as well as farmers and villagers who visited and participated in mass forums.

## 4. Results

### 4.1. Construction of Grassroots Water Conservancy Service Evaluation in Hunan Province

**Figure 2:**
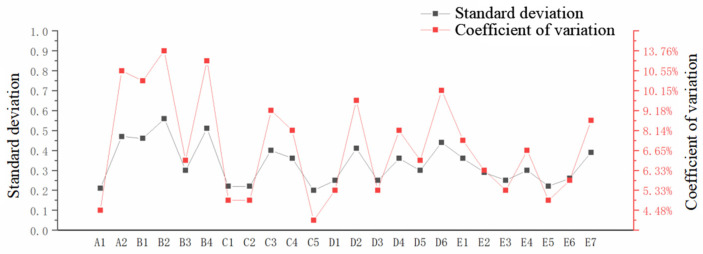
Standard deviation and coefficient of variations in index layer.

Indicators and weights were determined according to the methods described in Section 2.1 and Section 2.2. According to the construction principle of the evaluation index system, the main factors that affect grassroots water conservancy and the accessibility of data, this paper comprehensively constructs an evaluation index system of grassroots water conservancy service capacity in Hunan Province using five criteria: organizational structure, personnel quality, management level, public policy, and service capacity. By using qualitative and quantitative comprehensive evaluation methods. In total 24 representative indicators that reflect the current situation of the service capacity of various cities and prefectures in Hunan Province are selected; see Table 1 for specific indicators and algorithms. 24 indicators of the indicator layer were analyzed, 48 valid questionnaires were issued, and expert authority coefficients of the questionnaires were greater than 0.7. The final analysis results are shown in Figure 2 and Table 2. The average scores of final results are more than 4, the standard deviations are less than 0.57, the expert group’s indicators approval degree fluctuates slightly, and the coefficient of variation is less than 14%. Experts generally agree on the necessity of the index level of the evaluation index system, and it is feasible to implement the evaluation accordingly. The calculation results are shown in Table 3.

### 4.2. Evaluation of Water Conservancy Service Level in Hunan Province

**Figure 3:**
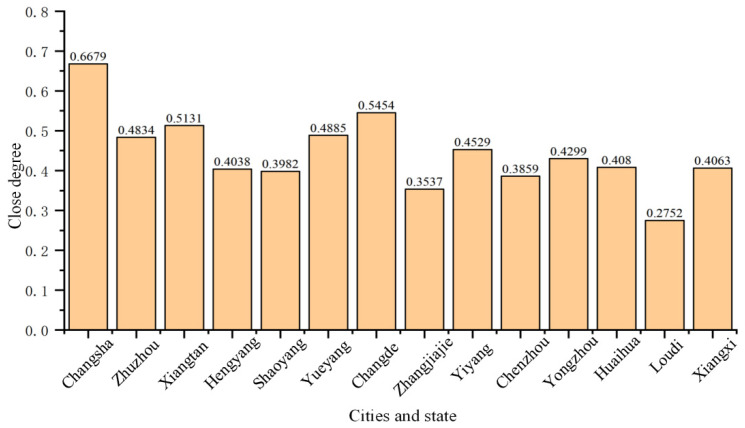
Result of closer degree of each city in Hunan Province.

All data were normalized based on the established evaluation model of grassroots water conservancy services in Hunan Province, and the positive ideal solution, negative ideal solution, and comprehensive closeness of grassroots water conservancy services in 14 cities and prefectures in Hunan Province were obtained using the gray correlation TOPSIS method model; see Table 4 for specific values. Figure 3 shows the closeness of each city and prefecture.

Based on the final evaluation and analysis results, the grassroots water conservancy services of cities and prefectures in Hunan Province are ranked from large to small as follows:

Chansha > Changde > Xiangtan > Yueyang > Zhuzhou > Yiyang > Yongzhou > Huaihua > Xiangxi > Hengyang > Shaoyang > Chenzhou > Zhangjiajie > Loudi. The comprehensive closeness Q value can be divided into the following three cases:

Category I: Q > 0.5 represents excellent water conservancy service at the grassroots level.

Category II: 0.4 < Q < 0.5 represents a good water conservancy service level at the grassroots level.

Category III: Q < 0.4 represents the average water conservancy service level at the grassroots level.

## 5. Discussion

### 5.1. Analysis of Evaluation Results

#### Criterion Level Analysis

In order to further analyze the specific situation of each city and prefecture, dimensions of the criteria level are analyzed. See Table 5 for specific data.

**Figure 4:**
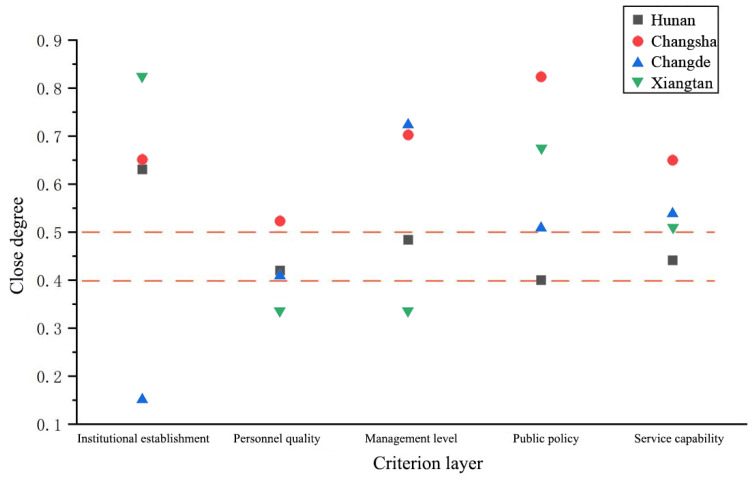
The first level of close degree of criterion layer of grassroots water service in Hunan Province.

It can be seen from Table 5 that the comprehensive closeness Q of the five evaluations in Changsha City is greater than 0.5, which is within an excellent range. In addition, public policy evaluation and service capacity evaluation are far higher than those of other cities and prefectures classified as Level I, and the service level of water conservancy at the grassroots level is significantly higher than that of other regions. The remaining dimensions of Changde City are higher than the average level of the whole province except for the evaluation of its organizational structure. However, Changde’s institutional reform has not yet been promoted yet. Its reform level ranks at the bottom of the whole province, and its comprehensive closeness is only 0.1429. Evaluation of the organizational structure of Xiangtan City is as high as 0.8189, which is in the upper reaches of the province. The other four dimensions fluctuate at the average level of the province. According to Figure 4, cities and prefectures at the first level of service have at least three evaluations above the provincial level, and one evaluation is at the top of the province. According to the value of comprehensive closeness, at least three evaluations have excellent comprehensive closeness.

**Figure 5:**
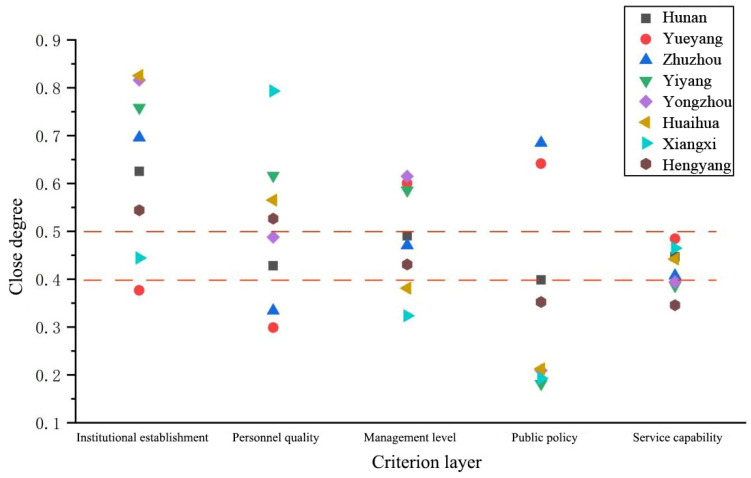
The second level of close degree of criterion layer of grassroots water service in Hunan Province.

As can be seen from Figure 5, cities and prefectures with secondary service levels share a common ground. The comprehensive closeness Q of service capacity evaluation is in the range of 0.4–0.5, and the service capacity is in a good stage. However, in other evaluations, each city or prefecture has one or two evaluations below the provincial average, and there is an obviously weak dimension in this regard. In Xiangxi Prefecture, the comprehensive closeness of the personnel quality evaluation dimension is as high as 0.7917, but the management level evaluation and public policy evaluation are only 0.3219 and 0.1911, respectively.

**Figure 6:**
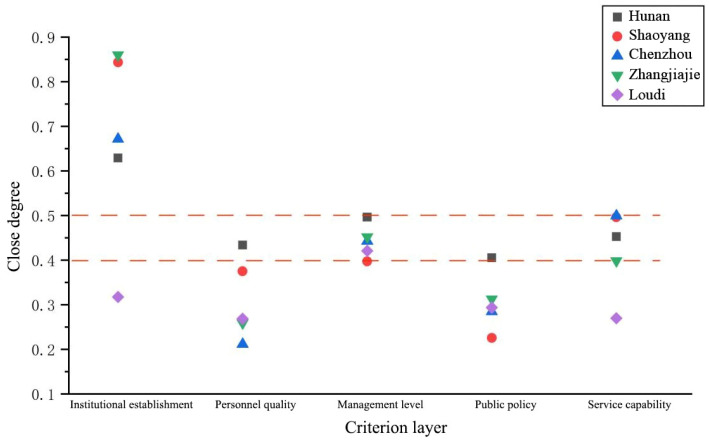
The third level of close degree of criterion layer of grassroots water service in Hunan Province.

It can be seen from Figure 6 that there are obvious weaknesses in cities and prefectures with three service levels. Barring the evaluation of institutional establishment and service capacity, the comprehensive closeness Q of the other three dimensions is below the provincial average. In addition, in the evaluation of personnel quality and public policy, the comprehensive closeness Q of cities and prefectures with three service levels is less than 0.4, which is at a general level.

### 5.2. Analysis of Obstacle Factors

**Figure 7:**
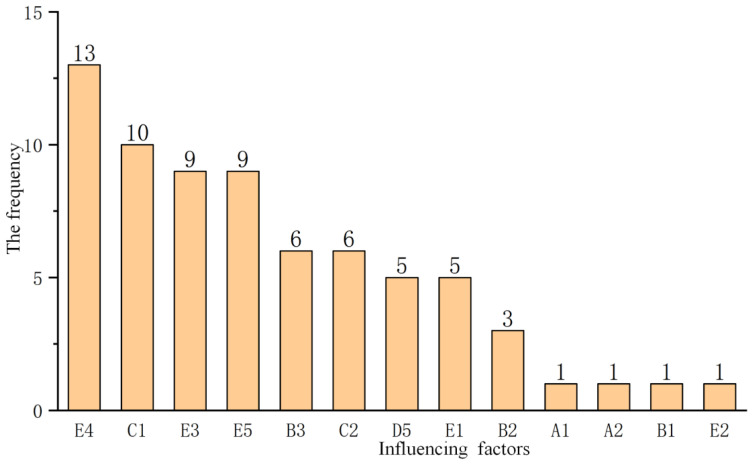
The frequency of main obstacle factors of each city in Hunan Province.

In order to analyze specific influencing factors, the top five obstacle factors are sorted according to the obstacle degree of individual indicators; these are then analyzed further to identify the weak points of grassroots water conservancy in cities and prefectures of Hunan Province, as shown in Table 6. The specific total frequency is shown in Figure 7.

After sorting out, the barrier factors E4 (urban and rural water supply capacity), C1 (personnel funding arrangement), E3 (irrigation water-saving capacity), E5 (embankment compliance capacity), B3 (professional water conservancy related proportion), and C2 (maintenance funding arrangement) were all indicators with a total frequency of more than 5 in the top five cities and prefectures.

It can be seen from Table 6 and Figure 7 that urban and rural water supply capacity, irrigation water-saving capacity, and embankment compliance capacity are indicators of grassroots water conservancy service capacity. There is still room for the development of grassroots water conservancy service capacity in Hunan Province. Urban and rural water supply capacity is an important part of building a moderately prosperous society and a solution to overcoming poverty. Except for Xiangtan, the irrigation water-saving capacity of other cities and prefectures ranks among the top five obstacle degrees. It is urgent to accelerate the construction of urban and rural water supply integration and solve the problem of uneven distribution of urban and rural water resources, water shortage in some areas, and seasonal water shortage.

**Figure 8:**
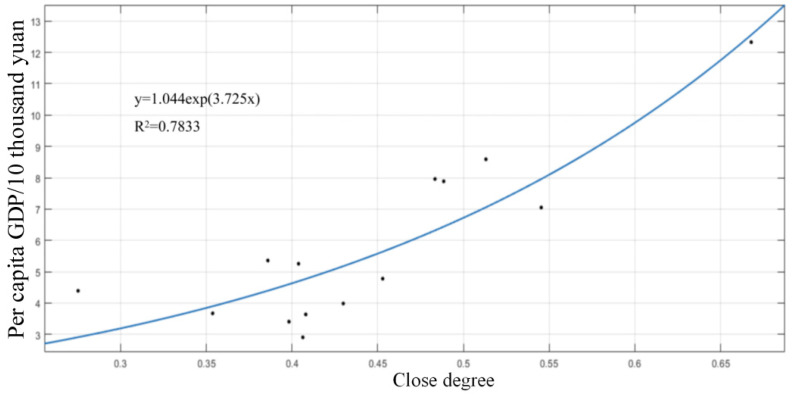
Relationship between per capita GDP and closer degree of grassroots water services in Hunan Province.

Personnel fund arrangements and maintenance fund arrangements are indicators of management level. Capital investment is an important pillar of water conservancy services at the grassroots level and an important factor restricting the management and service capacity of water conservancy institutions; moreover, it is also the basis and premise for forming their capacity to provide various water conservancy services, and thus it profoundly affects the overall level of water conservancy service capacity at the grassroots level. The realization of the supply function of grassroots water conservancy services and the improvement of public service capacity cannot be separated from the strong financial support of governments at all levels. Improving the institutional construction and the guarantee of personnel funds are the internal requirements for improving the capacity of grassroots water conservancy services and ensuring the effective supply of various public water conservancy services, as well as the key to improving the management level of grassroots water conservancy services. It can be seen from Figure 8 that the basic water conservancy service level in Hunan Province is quite similar to the local economic level. The more developed the economy, the higher the basic water conservancy service level. 

The proportion related to professional water conservancy belongs to the indicator of personnel quality dimension. As the carriers and practitioners, people are the basis of all systems. From the subjective factor of people, talents are key to improving grassroots water conservancy services and management abilities. The service capacity building of grassroots water conservancy institutions should ultimately be implemented toward cadres and front-line workers who perform various service functions. Improving the serviceability and management level of grassroots water conservancy institutions also tests the service awareness, service attitude, service skills, service means, and service specialization levels of staff. As the most intuitive embodiment of the service management level of grassroots water conservancy institutions, the serviceability of water conservancy cadres and ordinary employees directly determines the overall service management level of grassroots water conservancy institutions.

## 6. Conclusions

Based on the evaluation model of the grey correlation-TOPSIS method and the actual situation of Hunan Province, this paper proposes the evaluation index system of water conservancy services at the grassroots level in Hunan Province. The empirical study shows that:

1. The grey correlation TOPSIS evaluation model can be effectively used for the evaluation of grassroots water conservancy services in Hunan Province. In recent years, increased attention has been paid to the construction and thinking of grassroots water conservancy, but the evaluation index system—specifically for grassroots water conservancy—has not been studied. This study compares the construction process of the index system related to water conservancy modernization and highlights the government-leading nature of grassroots water conservancy in Hunan Province.
2. The subjective and objective comprehensive weighting method is used to determine the weight of evaluation indicators. This approach not only considers the subjective information of evaluation experts but also reflects the objective information of each indicator. Among them, service capability evaluation > management level evaluation > public policy evaluation > personnel quality evaluation > organization establishment evaluation. Service capability evaluation has the highest weight, 0.3453, which indicates that service capability has the greatest impact on the whole system.
3. The gray correlation TOPSIS method is used to evaluate the model, and the comprehensive closeness Q value is used to quantify the water conservancy service capacity level at the grassroots level in Hunan Province. The service capacity is classified into three levels: Changsha, Changde and Xiangtan are the first-level service capabilities; Yueyang, Zhuzhou, Yiyang, Yongzhou, Huaihua, Xiangxi and Hengyang are the second-level service capabilities; and Shaoyang, Chenzhou, Zhangjiajie and Loudi are the third-level service capabilities.
4. By fitting the comprehensive closeness and GDP data of cities and prefectures, the results show that there is considerable convergence between the grassroots water conservancy service level in Hunan Province and the local economic level; the more developed the economy is, the higher the basic water conservancy service level is. The realization of the supply function of grassroots water conservancy services and the improvement in public service capacity cannot be separated from the strong financial support of governments at all levels. Improving the institutional construction and personnel funding guarantees are internal requirements for improving the grassroots water conservancy service capacity and ensuring the effective supply of various public water conservancy services, but they are also key to improving the level of grassroots water conservancy services.
5. According to obstacle factor analysis, the service capacity, capital investment, talent construction and other factors of most cities and prefectures in Hunan Province are the key aspects restricting the development of grassroots water conservancy in Hunan Province. In the future development of grassroots water conservancy, cities and prefectures should establish a dynamic management system for obstacle factors, improve service capacity, increase capital investment, speed up talent development, reduce their obstacle level, and help rural revitalization.
6. The construction of the Hunan Province grassroots water conservancy services evaluation index system can effectively improve this province’s development of grassroots water resources. This is conducive to promoting the efficiency of grassroots water management in Hunan Province and also lays a solid theoretical foundation for Hunan Province to effectively and efficiently carry out its work by providing a guarantee for the sound operation of water resources in the grassroots sector.
7. The results of this study will be crucial in the discussion of how to improve the grassroots water service capacity in the future. In addition, the model of this study will be applied to other regions of China in subsequent studies to verify the generality of the model.

## Figures and Tables

**Table 1:** Selection and characterization of evaluation index.

Goal Layer	Criterion Layer	Index Layer	Index Type		Index Calculation Method
Grassroots water services in Hunan Province	A Organizational establishment evaluation	A1 Reform level of water conservancy management organization	Quantitative indicator	Forward	The proportion of administrative or public institution reform with staffing and financial arrangements
		A2 Proportion of comprehensive agricultural service centers	Quantitative indicator	Forward	Proportion of public institutions set up at the lowest level to directly serve “agriculture, rural areas and farmers”
	B Personnel quality evaluation	B1 Age structure of personnel	Quantitative indicator	Forward	Proportion of water conservancy workers under the age of 45
		B2 Education degree	Quantitative indicator	Forward	Proportion of personnel with professional education in total population of water conservancy
		B3 Proportion related to professional water conservancy	Quantitative indicator	Forward	Number of people related to water conservancy accounts for the proportion of people with professional education
		B4 Professional title level	Quantitative indicator	Forward	Proportion of personnel with professional titles in total staff of water conservancy
	C Management level evaluation	C1 Arrangement of personnel funds	Quantitative indicator	Forward	Sum of full appropriation and difference appropriation
		C2 Arrangement of Maintenance fund	Quantitative indicator	Forward	Arrangement of funds for maintenance and repair of water conservancy projects, and maintenance of machines, roads, buildings and other good conditions
		C3 Proportion of fixed office space	Quantitative indicator	Forward	Proportion of fixed office space in all management station buildings
		C4 Proportion of flood control warehouse	Quantitative indicator	Forward	Proportion of the number of flood control and drought relief warehouses in all management station buildings
		C5 Level of performance appraisal method	Qualitative indicators	Forward	Reflection
	D Public policy evaluation	D1 Supporting conditions of policies and regulations	Qualitative indicators	Forward	Score values of questionnaires
		D2 Construction level of technology promotion system	Qualitative indicators	Forward	Score values of questionnaires
		D3 Degree of government support	Qualitative indicators	Forward	Score values of questionnaires
		D4 Level of publicity and education	Qualitative indicators	Forward	Score values of questionnaires
		D5 Sound level of incentive mechanism	Qualitative indicators	Forward	Score values of questionnaires
		D6 Planned water use level	Qualitative indicators	Forward	Score values of questionnaires
	E Service capability evaluation	E1 Control capacity of water and soil loss	Quantitative indicator	Forward	Comprehensive control area of water and soil loss
		E2 River course regulation capacity	Quantitative indicator	Forward	Length of river reach reaching the standard
		E3 Water saving capacity of irrigation	Quantitative indicator	Forward	Areas of water-saving irrigation
		E4 Urban and rural water supply capacity	Quantitative indicator	Forward	Annual water supply designed for engineering design of urban and rural water supply
		E5 Embankment compliance capacity	Quantitative indicator	Forward	Length of qualified embankment
		E6 Effective utilization coefficient of irrigation water	Quantitative indicator	Forward	Ratio of water available for crops to total water used for irrigation
		E7 Water consumption per CNY 10000 of GDP	Quantitative indicator	Reverse	Ratio of annual water consumption to annual GDP

**Table 2:** Result of statistical analysis of indicators at the index layer.

Index Layer		Average	Standard Deviation	Coefficient of Variation
A1	Reform level of water conservancy management organization	4.71	0.21	4.48%
A2	Proportion of comprehensive agricultural service centers	4.42	0.47	10.55%
B1	Age structure of personnel	4.39	0.46	10.50%
B2	Degree of education	4.08	0.56	13.76%
B3	Proportion related to professional water conservancy	4.61	0.30	6.50%
B4	Level of professional title	4.08	0.51	12.43%
C1	Arrangement of personnel funds	4.68	0.22	4.74%
C2	Arrangement of Maintenance fund	4.68	0.22	4.74%
C3	Proportion of fixed office space	4.37	0.40	9.18%
C4	Proportion of flood control warehouse	4.45	0.36	8.14%
C5	Level of performance appraisal method	4.74	0.20	4.20%
D1	Supporting conditions of policies and regulations	4.61	0.25	5.33%
D2	Construction level of technology promotion system	4.42	0.41	9.33%
D3	Degree of government support	4.61	0.25	5.33%
D4	Level of publicity and education	4.47	0.36	8.14%
D5	Sound level of incentive mechanism	4.61	0.30	6.50%
D6	Planned water use level	4.32	0.44	10.15%
E1	Control capacity of water and soil loss	4.42	0.36	8.11%
E2	River course regulation capacity	4.63	0.29	6.33%
E3	Water saving capacity of irrigation	4.61	0.25	5.33%
E4	Urban and rural water supply capacity	4.58	0.30	6.65%
E5	Embankment compliance capacity	4.68	0.22	4.74%
E6	Effective utilization coefficient of irrigation water	4.53	0.26	5.66%
E7	Water consumption per CNY 10,000 of GDP	4.34	0.39	9.06%

**Table 3:** Weights of the evaluation index system of grassroots water services in Hunan Province.

Criterion Layer	Combined Weights	Index Layer	Entropy Method Weights	Analytic Hierarchy Process Weights	Combined Weights
A	0.0728	A1	0.0589	0.0190	0.0390
		A2	0.0406	0.0270	0.0338
B	0.1699	B1	0.0418	0.0320	0.0369
		B2	0.0374	0.0480	0.0427
		B3	0.0857	0.0430	0.0644
		B4	0.0279	0.0240	0.0260
C	0.2267	C1	0.0964	0.0600	0.0782
		C2	0.0728	0.0420	0.0574
		C3	0.0225	0.0120	0.0173
		C4	0.0460	0.0150	0.0305
		C5	0.0507	0.0360	0.0434
D	0.1854	D1	0.0163	0.0220	0.0192
		D2	0.0237	0.0350	0.0294
		D3	0.0270	0.0390	0.0330
		D4	0.0106	0.0390	0.0248
		D5	0.0271	0.0680	0.0476
		D6	0.0131	0.0500	0.0316
E	0.3453	E1	0.0766	0.0300	0.0533
		E2	0.0377	0.0460	0.0419
		E3	0.0408	0.0730	0.0569
		E4	0.0547	0.1030	0.0789
		E5	0.0415	0.0750	0.0583
		E6	0.0312	0.0250	0.0281
		E7	0.0190	0.0370	0.0280

**Table 4:** Evaluation results of grassroots water services in Hunan Province.

	Positive Ideal Solution	Negative Ideal Solution	Closeness Degree	Order by Closeness Degree
Xiangtan	0.3637	1.0000	0.6679	1
Hengyang	0.5894	0.5313	0.4834	5
Shaoyang	0.6158	0.6889	0.5131	3
Yueyang	0.6781	0.3759	0.4038	10
Changde	0.7233	0.4297	0.3982	11
Zhangjiajie	0.5457	0.5496	0.4885	4
Yiyang	0.4618	0.7639	0.5454	2
Chenzhou	0.9398	0.3229	0.3537	13
Yongzhou	0.6325	0.5855	0.4529	6
Huaihua	0.6997	0.3245	0.3859	12
Loudi	0.6052	0.4305	0.4299	7
Xiangxi	0.7614	0.4409	0.4080	8
Xiangtan	1.0000	0.1557	0.2752	14
Hengyang	0.7652	0.5156	0.4063	9

**Table 5:** Result of closer degree of criterion layer of each city in Hunan Province.

	Institutional Establishment	Personnel Quality	Management Level	Public Policy	Service Capability
Changsha	0.6439	0.5213	0.7004	0.8301	0.6402
Zhuzhou	0.6943	0.3326	0.4687	0.6828	0.4059
Xiangtan	0.8189	0.3447	0.3493	0.6775	0.5080
Hengyang	0.5425	0.5247	0.4289	0.3506	0.3439
Shaoyang	0.8406	0.3663	0.3885	0.2152	0.4891
Yueyang	0.3749	0.2969	0.5984	0.6394	0.4826
Changde	0.1429	0.4134	0.7206	0.5084	0.5367
Zhangjiajie	0.8571	0.2489	0.4444	0.3031	0.3900
Yiyang	0.7565	0.6147	0.5841	0.1798	0.3848
Chenzhou	0.6664	0.2008	0.4349	0.2752	0.4923
Yongzhou	0.8143	0.4863	0.6132	0.2076	0.3926
Huaihua	0.8237	0.5634	0.3795	0.2108	0.4404
Loudi	0.3083	0.2587	0.4127	0.2842	0.2601
Xiangxi	0.4427	0.7917	0.3219	0.1911	0.4631
Hunan Province	0.6234	0.4260	0.4890	0.3968	0.4450

**Table 6:** The main obstacle factors and the degree of obstruction in index layer of each city in Hunan Province.

	Factor 1	Obstacle Degree	Factor 2	Obstacle Degree	Factor 3	Obstacle Degree	Factor 4	Obstacle Degree	Factor 5	Obstacle Degree
Changsha	E4	22.20%	B3	16.37%	C1	15.88%	E1	9.72%	E5	8.24%
Zhuzhou	E4	14.00%	C1	12.60%	E5	12.60%	B1	7.13%	E1	7.12%
Xiangtan	C1	13.50%	E5	11.44%	E3	10.48%	E1	10.47%	B3	9.06%
Hengyang	E4	11.23%	C1	10.89%	E3	8.68%	E5	7.59%	D5	6.51%
Shaoyang	E4	11.46%	C1	9.55%	E5	8.93%	C2	6.89%	D5	6.73%
Yueyang	E4	14.12%	E3	8.49%	B2	7.61%	E1	7.48%	B3	6.98%
Changde	E4	13.32%	E3	8.81%	A1	8.05%	A2	6.99%	B2	6.93%
Zhangjiajie	E4	11.86%	C1	11.76%	B3	9.56%	C2	8.63%	E3	8.56%
Yiyang	E4	13.32%	E3	8.63%	D5	7.35%	E2	7.27%	E1	7.21%
Chenzhou	E4	11.29%	C1	10.50%	B3	8.92%	B2	6.89%	C2	6.44%
Yongzhou	E4	11.94%	C2	8.17%	E3	7.89%	E5	7.14%	D5	6.76%
Huaihua	E4	12.31%	C1	11.39%	C2	7.87%	E5	7.87%	D5	7.78%
Loudi	E4	9.66%	C1	8.97%	B3	8.53%	E5	7.66%	E3	7.03%
Xiangxi	C1	11.73%	E4	10.11%	E5	8.25%	E3	8.04%	C2	7.97%

## Data Availability

The data presented in this study are available on request from the corresponding author Bin Deng, at dengbin07@csust.edu.cn. The data are not publicly available in accordance with participant privacy.
